# Do not blame the organizational culture for poor performance

**DOI:** 10.1093/intqhc/mzaf092

**Published:** 2025-09-19

**Authors:** Dinesh K Arya

**Affiliations:** University of Tasmania, 80 Argyle St, Hobart, Tasmania, 7000, Australia

**Keywords:** culture, performance

## Abstract

The formal and informal ways in which things are done in an organisation explain the behaviours that guide activities in that organisation’s environment, or in other words, the culture of the organisation. When things go wrong, it is not unusual for an organisation’s culture to be implicated as ′bad’ and labelled as the reason for inadequate or underperformance.

The underlying supposition in making the culture of an organisation an issue is to make a case that a change in the culture will, in some way, improve organisational performance. Programs to effect this change are implemented, despite little evidence that a change in organisational culture will result in different performance or outcomes. Moreover, more often than not, strategies implemented to deliver cultural change do not change the culture of an organisation.

## Introduction

The values and beliefs that are commonly held, the norms that evolve and the behaviours that result determine an organization’s culture [1]. Since culture correlates with organizational performance, many leaders see changing culture as a recipe for improving performance [2]. Indeed, it is not unusual for an incoming Chief Executive to blame the ‘prevalent culture’ for underperformance or to make a case for change of culture [3]. Since the culture of an organization is not easy to describe or define, nor is change easy to measure, the proposition that culture is to blame for underperformance cannot be easily challenged.

Even though there is little evidence that leaders can influence and change the culture of an organization, and many studies suggest the evidence of influence of organizational culture on performance of an organization is limited, conflicting, and inconclusive [4–6], the proposition that leaders can change the culture and therefore improve organizational performance remains attractive (e.g. Schein’s multi-layered organisational culture model [7], Denison Organizational Culture Model [8], and Thomas and Peterson’s Theory of Organizational Excellence [9]). Perhaps this is because changing the culture of an organization takes a long time; it is extremely difficult to measure culture change, and even if nothing changes, a new leader can ask for the organization to be patient while the cultural change is in progress. Some may also see it as an opportunity to put their own stamp on how things should be done, which they believe would be good for the organization.

Some leaders (especially incoming Chief Executives) often fall into the trap of believing that their personal values or preferred ways of working represent a ‘better’ culture for the organization. This self-belief can create blind spots, making them less responsive to actual reasons for underperformance. While culture is important, it is complex, deeply rooted, and slow to change. Attempting to overhaul it based on subjective perceptions or vague ideals often leads to disruption without measurable improvement.

## Many different cultures exist in an organization

A common cause of organizational underperformance is misalignment between strategic direction and prevailing behaviours—not necessarily the culture itself. Moreover, it is too simplistic to consider the culture of organizations as a singular concept. Studies that have attempted to map the culture and identities within organizations have discovered that many different ideologies, identities, and therefore cultures exist at any given time [10]. This is often the reason for strength and resilience within those organizations.

For leaders to think that the top management can prescribe and direct a new or different culture is surprisingly common. This is despite the recognition that a multitude of ideas, beliefs, values, and behaviours evolve in an organization over a prolonged period and do not necessarily change easily. Moreover, various internal and external influences shape the diverse ideologies and cultures within an organization, and these cultures evolve in response to the stresses and strains they encounter. That is another reason why these are also resistant to change. In an evolutionary sense, this occurs to give the organization the best chance to survive. To then want to intervene and try to change it does not make sense.

If a case cannot be made to blame the culture for underperformance, the question then becomes what leaders can do when underperformance occurs.

### Consider the misalignment of culture with strategy

Once strategy and culture are broadly aligned, the next step is to examine behaviours that may have become maladaptive over time. These behaviours may not reflect the organization’s core values but may have emerged due to legacy practices, outdated incentives, or environmental shifts. For example, if the direction in which the organization needs to head is to achieve excellence or a higher level of collaboration or partnerships, behaviours that may be necessary to achieve this strategic direction, such as openness, accountability, or agility, may need to be developed, learnt or adopted. Crucially, behavioural change should be evidence-based. Leaders must identify behaviours that are clearly counterproductive or misaligned with strategic objectives. Presuming that the organization has the ‘wrong’ culture and that the leader knows the ‘right’ one is a fallacy. Underperformance is rarely caused by belief systems alone, and blaming culture without evidence does little to drive meaningful change.

### The strength and resilience of culture is something to celebrate

More often than not, the ‘new’ proposed cultural change is no different from the existing culture. Rather than disrupting this equilibrium with speculative cultural change, leaders should celebrate and build on these strengths. Supporting the culture while enabling behavioural shifts through clear policies, incentives, and leadership modelling is far more effective.

Supporting the organizational culture and facilitating adoption of the right behaviours, backed by necessary policies and strategies, is what results in enduring change. People need reasons to behave differently and have to be supported to make that change. The need for behavioural change, accompanied by clear evidence of counterproductive behaviours, can provide convincing evidence to justify the organization’s need to improve its performance.

Changes made must always be supported by ongoing monitoring to consider whether or not the behaviours and resulting performance are changing. Faulting the existing culture of the organization is often no more than an empty slogan that does not bring about change.

## Conclusions

Many cultures that exist within an organization are often the reason for its strength and resilience. If the performance of the organization has changed, implying the prevailing culture as the reason for underperformance is a mistake. Slogans that the culture of the organization needs changing are even greater fallacies when there is no evidence that culture change strategies result in either a change in culture or performance.

Leaders should avoid abstract cultural overhauls and instead focus on targeted behavioural change. This approach is practical, evidence-based, and aligned with strategic goals—ultimately leading to more meaningful and lasting improvements in organizational performance.

## Data Availability

Not applicable.
